# How Do Families of Children with Down Syndrome Perceive Speech Intelligibility in Turkey?

**DOI:** 10.1155/2015/707134

**Published:** 2015-04-21

**Authors:** Bülent Toğram

**Affiliations:** Anadolu University, Dilkom, Yunus Emre Kampusu, 26470 Eskisehir, Turkey

## Abstract

Childhood verbal apraxia has not been identified or treated sufficiently in children with Down syndrome but recent research has documented that symptoms of childhood verbal apraxia can be found in children with Down syndrome. But, it is not routinely diagnosed in this population. There is neither an assessment tool in Turkish nor any research on childhood verbal apraxia although there is a demand not only for children with Down syndrome but also for normally developing children. The study examined if it was possible to determine oral-motor difficulties and childhood verbal apraxia features in children with Down syndrome through a survey. The survey was a parental report measure. There were 329 surveys received. Results indicated that only 5.6% of children with Down syndrome were diagnosed with apraxia, even though many of the subject children displayed clinical features of childhood verbal apraxia. The most frequently reported symptoms of childhood verbal apraxia in literature were displayed by the children with Down syndrome in the study. Parents could identify childhood verbal apraxia symptoms using parent survey. This finding suggests that the survey can be developed that could serve as a screening tool for a possible childhood verbal apraxia diagnosis in Turkey.

## 1. Introduction

Intelligibility refers to the level that a listener understands acoustic signals produced by a speaker without any supporting information [1, 2]. Speech intelligibility difficulties are a frequent issue that arises at the early stages of speech production for children with Down syndrome (DS) [3–6] and it continues during both adolescence and adulthood [6–8]. The intelligibility level of individuals with DS is far lower than their cognitive age [9]. The effectiveness of their communication with the environment is highly dependent on the level of speech intelligibility even if the expressive language skills of children with DS develop in time [10]. Whereas normally developing children master speech intelligibility within 48 months, intelligible speech production prevails as a major difficulty for individuals with DS throughout their lives [11]. Literature hosts many studies concluding that low level of speech intelligibility is a common problem for individuals with Down syndrome [4, 6, 7, 10, 12–17].

The speech production of many individuals with DS is impaired due to several motor speech difficulties stemming from inadequate central motor control and failure in programming, combining, arranging, and sequencing the fine motor movements necessary for proper and exact articulation [18]. Besides, speech production is also affected by some anatomic features specific to individuals with DS [11, 19–21]. Articulation movements can be negatively influenced by several factors such as smaller oral cavity than normal (causing the perception of a huge tongue), hypotonic muscles around the mouth, joint lip muscles, and excessive amount of lip muscles. These differences in structure and in tongue size influence the production of lingual consonants. Furthermore, weak facial muscles limit lip movement, thus affecting production of labial consonants and rounded vowels. General hypotonicity affects lip and tongue movements involved in all aspects of speech production. Any one of these factors is likely to influence motor movements associated with speech and negatively impact the articulatory and phonatory abilities of children with Down syndrome [17]. Furthermore, different neural innervation also impedes the speed and variety of speech movements [7]. All these anomalies distort the intelligibility and speech of individuals with DS. Regarding the anatomical condition, individuals with DS generally have weak motor function performances [19, 22, 23], and they are known to display a specific deficiency in motor control especially during speech production [6]. Barnes, Roberts et al. [24] state that boys with DS significantly differ from their normally developing peers having corresponding nonverbal cognitive age in terms of the functions of tongue, lips, velopharynx, larynx, and coordinated speech. Similarly, individuals with DS were also identified to have lower levels of coordinated speech movements as opposed to boys with Fragile X with matching nonverbal cognitive age. In Kumin's study [6], 58% (frequently) of families say that strangers do not understand their children's speech and 37% say that their children's speech is not understood by sometimes.

McCann and Wrench [25], in children with DS, examined diadochokinetic (DDK) skills and found low levels of accuracy in the /ptk/ sequencing in their study. This finding pointed that dyspraxia should be an important diagnosis option to be considered for individuals with DS. Kumin et al. [4] report that there are motor and sensory deficits caused by a cognitive impairment and necessary for motor planning in the speech of children with DS. They also note that this situation causes some behavioral features of apraxia to surface making speech more difficult to understand as the utterance gets longer and that difficulties in sequencing and grouping are observed.

There are two factors affecting speech intelligibility. These are oral-motor (OM) skills and oral-motor planning (OMP) [4, 15, 16, 26–28]. Oral-motor skills refer to strength and the movement of the muscles of the face (e.g., mouth, jaw, tongue, and lips). This includes muscle tone, muscle strength, range of motion, speed, coordination, and dissociation (the ability to move oral structures, such as the tongue and lip, independently of each other) [29]. The acquisition and maturation of oral-motor movements underlie sound production and feeding skills (e.g., sucking, biting, and chewing) [30]. Motor planning is the process of deciding what your body has to do and then doing it. Motor planning refers to process that includes conceiving, planning, sequencing, and executing actions [31]. OMP refers to skills of combining and sequencing speech sounds to articulate words [5]. One of the problems that may occur during OMP is called childhood verbal apraxia (CVA). Among the common features of this speech problem reported in the literature are inconsistency in sound production [32, 33], reduced intelligibility in utterance length [32, 34], a limited repertoire of sounds [34, 35], difficulty in imitation [33, 34], difficulty combining and sequencing sounds [32, 35], sound and syllable reversals [34, 35], speech rhythm difficulties [34, 36], and struggle on production of speech and/or nonspeech tasks [32, 34, 35]. Furthermore, Dodd and Thompson [37] clearly show that children with DS are inconsistent in speech production, with over half of all words produced being pronounced differently on repeated productions. But, the problems are called as inconsistent phonological (speech) disorder. The disorder may explain that speech characterized by variable productions of the same lexical items or phonological features is not only from context to context but also within the same context. Inconsistency characterized by multiple error types (unpredictable variation between a relatively large number of phones) suggests the lack of a stable phonological system because of a deficit in phonological planning. Phonological planning refers to the process of phoneme selection and sequencing [38]. Inconsistent speech disorder is distinct from childhood apraxia of speech (CVA), although inconsistency characterizes both disorders [39].

It is also known that most children with DS display apraxic features, but this disorder is rarely diagnosed together with DS [40]. Related literature contains numerous studies concluding that children with DS have difficulties in oral-motor skills, oral-motor planning, and both at the same time [4, 15, 16, 26–28, 41].

In their study “The Apraxia Profile” conducted on seven children with DS using a family scale and conversation based speech sample, Kumin and Adams [4] reported that children with DS exhibited CVA features and that these features were the same as those displayed by children diagnosed with CVA who are normally developing cited in the literature.

Assessment and therapy methods regarding normally developing children with CVA in the literature are also employed for children with DS. However, Kumin [5] developed the Down Syndrome Speech Intelligibility Survey because there had been no evaluation procedure to diagnose CVA and applied it on the parents of 1620 children with DS. At the end of the study, parents were determined to identify CVA features exhibited by their children through using the Survey. Roberts et al. [11] revealed that speech apraxia and dysarthria features were observed on individuals with DS. These findings underpin that the assessment of muscular tone and speech coordination is crucial for the planning of the treatment. Thus, identifying the difficulties in OM skills and the existence of CVA in individuals with DS has drawn a lot of attention in the literature recently.

On the contrary, there is neither an assessment tool in Turkish nor any research on OM difficulties or CVA although there is a huge demand not only for individuals with DS but also for normally developing children. There is a need for more research in order to understand the nature of speech disorders observed in individuals with DS and to design appropriate therapy methods. This research examined if it was possible to determine OM difficulties and CVA features in children with DS through a survey about family opinions.

## 2. Method

In this research, parents were asked to describe speech properties of their children with DS by responding to Down Syndrome Speech Intelligibility Survey developed by Kumin [5] and translated and adapted into Turkish by the researchers. Having 40 items to be answered on a four-scale Likert type grading, ranging from “Always,” “Frequently,” “Sometimes,” to “Never,” the survey was administered on 329 family members/parents of children with Down syndrome continuing to a private special education and rehabilitation center in either Istanbul, Eskisehir, Ankara, Izmir, or Gaziantep. Inclusion of the participants to the study was based on willingness. Families were asked to answer the survey items related to their children's speech.

### 2.1. Adaptation of the Survey to Turkish Language

The survey including 40 items was translated from English into Turkish by 3 speech and language pathologists (SLPs). The three forms of the survey which was translated from English into Turkish were examined by 7 SLPs who have doctorate degree and accuracy of translation of every item in the survey from English into Turkish was evaluated. The SLPs examined translation of every item upon 3 translation forms and marked the most accurate translation for every item on the evaluation form. Then, with the data obtained from the SLPs, a decision was made on which translation was appropriate for every item (kappa coefficient 0.98). Therefore, the content of every item was determined, necessary arrangements were made, and the final form of the survey to be used in the study was prepared by the researcher. The items of the survey in Turkish version were the same as in the original one.

### 2.2. Demographic Information on the Children with DS

The number of boys outnumbered the girls; 63.3% (*n* = 202) were boys and 36.7% (117) were girls. The age range of children was between 1 and 19 years with an average of 5.3 years (SD = 3.7). When asked about the onset of speech in their children, 26.3% of families answered 2 years onwards, 17.6% said 3 years onwards, 14.1% replied 4 years onwards, 6% stated 5 years onwards, and 3.1% answered 6 years onwards. Moreover, 32.9% of families stated that their children did not speak (mean: 2.4 yrs. SD: 1.4).

The percentages of families whose children were diagnosed with OM difficulty and CVA were 37.9% (*n* = 121) and 5.6%, respectively. According to their families' statements, speaking (47% *n* = 150), mimes and gestures (46.7% *n* = 149), and others (6.3% *n* = 20) were among the communication forms used by children with DS. All pieces of information about the children with DS were obtained from the family survey.

### 2.3. Data Analysis

Data were processed on SPSS 17, and statistical analyzes were run. The frequency of answers provided for each item was calculated, and the percentages of the items regarding symptoms of childhood verbal apraxia and speech intelligibility were determined.

## 3. Results

Table 1 depicts the frequencies and percentages of the answers given by the families concerning their children with DS.

### 3.1. Speech Intelligibility

Within the survey, families were asked to rate their children's speech between 1 and 10 (1 being totally unintelligible and 10 being totally intelligible), and the average was 4.2 (SD = 2.7). Findings regarding speech intelligibility ratings can be found in Table 2.

### 3.2. The Relation between Speech Intelligibility and Gender and Age

The relation between families' speech intelligibility rating and gender and age was analyzed through Pearson correlation. Age and speech intelligibility were determined to have a significant level of correlation (*r* = 0.317, *P* < 0.01); and older children were reported to have better levels of speech intelligibility. Likewise, another significant correlation was established between speech intelligibility and gender (*r* = −0.143, *P* < 0.05), girls being more intelligible than boys.

### 3.3. Oral-Motor Skills

It is common knowledge that children with DS experience difficulties with OM skills. In this study as well, 37.9% of families stated that their children were diagnosed with OM difficulty. Analysis of the answers concerning OM skills showed that 39.8% of children experienced problems with OM skills. Children were reported to have had less muscular tone when they were babies and to have built stronger muscles in time. The percentage of children who were noted not to have lower levels of muscular tone when they were babies was 29.2 while 48.3% of children were recorded without any current low muscular tone (Table 3).

### 3.4. Childhood Verbal Apraxia

According to the information provided by the families, only 5.6% of children with DS were diagnosed with apraxia. No statistically significant correlation between CVA and age (*r* = −0.011, *P* > 0.01) or CVA and gender (*r* = 0.017, *P* > 0.01) was found. Besides, the correlation between speech intelligibility rating and CVA was studied, and those diagnosed with CVA were determined to have lower ratings of speech intelligibility with weak relationship (*r* = −0.115, *P* < 0.05).

### 3.5. Inconsistent Speech Production

One of the prominent features of CVA is inconsistent speech production.  Table 4 depicts the answers given by the families to the items regarding inconsistent speech production in the survey.

A large proportion of families said that their children had inconsistent speech errors. Families responses about the item “Sometimes, my child can say a word but at other times, my child has difficulty saying the same word” and the item “My child may unexpectedly say a word or phrase perfectly, but then s/he can't repeat it” were strikingly similar.

### 3.6. Increasing Length and Complexity

Another feature of CVA is that the intelligibility of speech decreases as the length and complexity of sentences increase. Findings concerning the four items on this in the survey are represented in Table 5.

Findings indicate that children with DS experience more difficulty as the words or phrases get longer and more complex.

### 3.7. Consonant and Vowel Production

Generally, children with CVA have a limited repertoire of speech sounds, and they are known to drop sounds or syllables in words and to make metathetic mistakes. Families' responses about consonant and vowel production are shown in Table 6.

As for families, most of children with DS go through troubles during vowel and consonant production, omit sounds or syllables in words, and change the place of sounds in a word.

### 3.8. Imitation Skills

Difficulty to imitate is a common symptom of childhood verbal apraxia. Families stated that their children with DS had hard times imitating through the answers they gave for one item in the survey (Table 7).

### 3.9. Prosody and Rhythm

Disorders in the prosody and rhythm of speech (sound lengthening, stress mistakes, etc.) are also other symptoms of childhood verbal apraxia. Two items in the survey are related to this property, and relevant findings are given in Table 8.

According to the information provided by the families, more than half of children with DS prolonged vowels and had difficulty in speaking fluently.

### 3.10. Struggle When Speaking

Struggling while producing sounds (trial and error) is accepted as one other indicator of CVA.  Table 9 presents relevant findings.

Almost 80% of children with DS were reported to struggle (exhibiting search behavior) while producing words.

### 3.11. Hearing Loss

According to the information provided by the families, almost all the children (90.3%) were reported not to have any hearing problems by their families (Table 10).

### 3.12. Relationship between Age of First Word and CVA

Families of children with DS and diagnosed with CVA stated that the onset of speech was late for their children (around 5 years). The relation between the age of first word and speech intelligibility rating was determined to be significant (significance > 0.01); hence, families of children who started speaking after 5 years of age gave lower ratings for the intelligibility of their children's speech.

### 3.13. Diagnostic Label of Difficulty with Oral-Motor Skills or CVA

Examination of the data provided by the families through the survey revealed that 32.3% of children had difficulty with OM skills; 5.6% of children had problems with OM skills and were diagnosed with CVA; and 62.1% of children were reported not to have any diagnoses. Therefore, 37.9% of children were noted to have OM difficulties and 5.6% of children were recorded to have CVA diagnoses.

## 4. Discussion

There is no test to assess CVA in Turkey. Thus, diagnosing verbal apraxia in both normally developing children and children with developmental delays stands as a major issue. This study examined if it was possible to identify difficulties in OM skills and CVA features exhibited by children with DS based on the families' opinions recorded through a survey. Findings showed that this survey could be used as a screening tool for a possible CVA diagnosis for Turkish speaking children with DS, consistent with those of Kumin [5]. Nevertheless, children with suspected CVA should be subjected to advanced clinic examination. This study reached some findings inconsistent with literature, but similar to those of Kumin [5]. Literature points that errors in verbal apraxia are inconsistent. In Kumin's study [5], total amount of “always—frequently—sometimes” responses given to the item “14” indicated that 79.3% of families thought the errors made by their children were consistent whereas the percentage in the present study was 91.8. Thus, it is believed that families answered this item by assuming that the item asked if their children made any speech errors or not.

Almost all the children (90.3%) were reported not to have any hearing problems by their families. Approximately two-thirds of children with DS experience conductive hearing loss, sensorineural hearing loss, or both [42]. Despite this information in the literature, the fact that the families did not mention any hearing loss may be pointing the need to investigate this with more specific questions in the survey as also underlined by Kumin [5]. In addition, the limitations with the procedures to diagnose hearing problems in Turkey should also be considered.

Almost 90% of children with DS are known to be unable to produce sounds during cooing and babbling stages. In spite of the fact that many studies indicate that children with DS do not differ from normally developing children in terms of early language development, some research concludes that children with DS start canonic babbling two months later (around nine months) than the other babies. Hypotonic muscular structure can be given as a reason for this [17]. Likewise, babies with DS do not experience any difficulties during sucking and swallowing both liquid and solid foods, and nearly half of the subjects were noted not to go through any troubles while eating; yet, 14.1% of them were reported to suffer from problems swallowing liquids. On the other hand, weakness in the facial muscles restricts their lip movements, and this impedes the production of labial consonants and rounded vowels. General hypotonicity of muscles impacts all lip and tongue movements related to speech negatively. All the aforementioned factors form a drawback on the motor movements of children with DS; hence, articulation and phonation skills of these kids are limited [17].

The children with Down syndrome almost always draw attention to the delays to be expected in their speech and language development. Despite a wide range of individual differences, most children are late in saying their first words; their vocabulary grows more slowly than in ordinary children [43, 44]. Average age when children with DS start speaking is 18 months, but this may vary across individuals. First words are generally names of people or animals. These children begin using two word utterances around 30 months [8]. According to family records, 90% of the children in the study had a delayed onset of speech, consistent with those of the literature.

Almost all the children (93%) were noted to omit sounds during speech in this study. Some of the phonological errors exhibited by boys with DS are reduction of consonant clusters, omission of the word-final consonants, decrease in using phrases, stopping, and reduction of liquids as mentioned by Bleile and Schwarz [45]; Dodd and Thompson [37]; Kumin [6]; Smith and Stoel-Gammon [46]; Stoel-Gammon [17]. As opposed to their normally developing peers, children with DS make more mistakes in consonant production and tend to distort the form of the syllables by omitting some of the parts or syllables more frequently; hence, the intelligibility of speech worsens [47]. As for families, nearly all the children were able to understand more than they produced. Language characteristics of school age children with DS show that their receptive vocabulary is relatively better, but their receptive syntax is lower than their nonverbal cognitive level. Findings revealed that boys with DS scored lower than their peers in comprehending sentences with more complex grammar and morphology. Similarly, receptive language of children at this age is not as high as expected in accordance with their cognitive age [8].

Compatible with the findings of Kumin [5], 87% of participating families mentioned that people familiar with their children had difficulties in understanding their children's speech and that almost everybody (94.7%) seeing their children for the first time did not understand the speech at all. When the children's speech is not comprehended, what happens most often (%95.3) is the help by family members. A great number of children (92%) had difficulties in producing long words whereas more (97%) were struggling during uttering longer sentences. On the contrary, 95.6% of children with DS were recorded to have higher levels of intelligibility during production of familiar words and phrases.

Moreover, the findings of this study are consistent with those of other studies in the literature in terms of common features of CVA (e.g., inconsistent speech production, imitation skills, and struggle when speaking (trial and error) [5, 32–36].

Cumulatively, findings pointed that the existence of CVA symptoms could be detected in children with DS and that families were able to identify CVA symptoms in their children through the survey. A serious problem with respect to diagnosing CVA in children with DS was also found. Even though many of the subject children displayed features of CVA, only 5.6% of them had been diagnosed with it. Because the number of certified speech and language pathologists (SLPs) in Turkey is limited, diagnosis of CVA is difficult not only in children with DS but also in normally developing children. Although the literature has a vast amount of research underpinning the importance of teaching speech sounds to children with CVA, there seems to be no therapy program either for early childhood or later ages for the benefit of children with DS due to the inadequate number of speech and language pathologists. Children with DS generally prefer using gestures and mimes over vocalizing speech sounds especially during the early stages of language development. Since using gestures is linked with psychomotor development and lexical comprehension, it is thought to be a compensation strategy for the difficulties in verbal production [48].

In the literature, hallmark features of CVA listed include (a) inconsistent errors on consonants and vowels in repeated productions of syllables or words, (b) lengthened and disrupted coarticulatory transitions between sounds and syllables, and (c) inappropriate prosody, especially in the realization of lexical or phrasal stress [49]. The 3 out of 4 key features can serve a CVA diagnosis. However, the conclusion that parents report is sufficient for screening of CVA in children with DS. Thus, in conclusion, the results of the study showed that this survey could be used as a screening tool for a possible CVA diagnosis for Turkish speaking children with DS. But, because children with DS have some other type of speech sound disorders, they need to be assessed by a SLP with standardized tests and clinical examinations and an accurate differential diagnosis.

## Figures and Tables

**Table 1:** Frequencies and percentages of responses to survey questions (see Appendix).

Number of Oues.	Always		Frequently		Sometimes		Never	
	*F*	%	*F*	%	*F*	%	*f*	%
1	62	19.4	45	14.1	171	53.6	41	12.9
2	120	37.6	90	28.2	92	28.8	17	5.3
3	87	27.3	66	20.7	94	29.5	72	22.6
4	98	30.7	82	25.7	124	38.9	15	4.7
5	105	32.9	93	29.2	89	27.9	32	10
6	122	38.2	82	25.7	77	24.1	38	11.9
7	37	11.6	37	11.6	71	22.3	174	54.6
8	49	15.4	24	7.5	114	35.7	132	41.4
9	3	0.9	0	0	42	13.2	274	85.9
10	25	7.8	18	5.6	100	31.3	176	55.2
11	65	20.4	62	19.4	99	31	93	29.2
12	24	7.5	33	10.3	108	33.9	154	48.3
13	192	60.2	44	13.8	52	16.3	31	9.7
14	122	38.2	53	16.6	118	37	26	8.2
15	73	22.9	34	10.7	159	49.8	53	16.6
16	118	37	70	21.9	108	33.9	23	7.2
17	78	24.5	64	20.1	118	37	59	18.5
18	78	24.5	67	21	103	32.3	71	22.3
19	124	38.9	44	13.8	95	29.8	56	17.6
20	58	18.2	52	16.3	144	45.1	65	20.4
21	17	5.3	33	10.3	88	27.6	181	56.7
22	33	10.3	23	7.2	80	25.1	183	57.4
23	12	3.8	8	2.5	11	3.4	288	90.3
24	113	35.4	92	28.8	88	27.6	26	8.2
25	148	46.4	96	30.1	66	20.7	9	2.8
26	71	22.3	74	23.2	94	29.5	80	25.1
27	55	17.2	53	16.6	113	35.4	98	30.7
28	65	20.4	87	27.3	128	40.1	39	12.2
29	83	26	46	14.4	118	37	72	22.6
30	40	12.5	16	5	113	35.4	150	47
31	104	32.6	85	26.6	108	33.9	22	6.9
32	101	31.7	69	21.6	109	34.2	40	12.5
33	33	10.3	15	4.7	48	15	223	69.9
34	80	25.1	53	16.6	126	39.5	60	18.8
35	88	27.6	58	18.2	116	36.4	57	17.9
36	176	55.2	83	26	46	14.4	14	4.4
37	229	71.8	48	15	39	12.2	3	0.9
38	63	19.7	60	18.8	160	50.2	36	11.3
39	141	44.2	75	23.5	84	26.3	19	6
40	65	20.4	31	9.7	163	51.1	60	18.8

**Table 2:** Speech intelligibility ratings of parents.

Score	Frequency	Percentage
1	72	22.6
2	46	14.4
3	34	10.7
4	25	7.8
5	44	13.8
6	23	7.2
7	37	11.6
8	13	4.1
9	6	1.9
10	19	6

Total	319	100

**Table 3:** Percentages of parents on questions of oral-motor skills.

	Always	Frequently	Sometimes	Never

(11) My child had low tone in the muscles of the face (lips, tongue, and cheeks) in infancy	20.4%	19.4%	31%	29.2%

(12) My child currently has low tone in the muscles of the face (lips, tongue, and cheeks)	7.5%	10.3%	33.9%	48.3%

**Table 4:** Percentages of parents on questions of inconsistent speech production.

	Always	Frequently	Sometimes	Never

(14) My child makes the same speech errors consistently	38.2%	16.6%	37.%	8.2%

(15) Sometimes, my child can say a word but at other times, my child has difficulty saying the same word	22.9%	10.7%	49.8%	16.6%

(38) My child may unexpectedly say a word or phrase perfectly, but then she/he cannot repeat it	19.7%	18.8%	50.2%	11.3%

**Table 5:** Percentages of parents on questions of increasing length and complexity.

	Always	Frequently	Sometimes	Never

(16) My child is understandable when she/he says single words but has greater difficulty in conversation	37%	21.9%	33.9%	7.2%

(24) My child has more difficulty saying longer words than shorter words	35.4%	28.8%	27.6%	8.2%

(25) My child has more difficulty speaking when she/he is using longer phrases or sentences	46.4%	30.1%	20.7%	2.8%

(36) My child's speech is easier to understand when she/he is saying familiar words	55.2%	26%	14.4%	4.4%

**Table 6:** Percentages of parents on questions of consonant and vowel production.

	Always	Frequently	Sometimes	Never

(26) My child has difficulty saying some consonant sounds	22.3%	23.2%	29.5%	25.1%

(27) My child has difficulty saying some vowel sounds	17.2%	16.6%	35.4%	30.7%

(28) My child often reverses sounds in words (e.g., aminal for animal)	20.4%	27.3%	40.1%	12.2%

(31) My child leaves out sounds in words	32.6%	26.6%	33.9%	6.9%

(32) My child leaves out syllables in words	31.7%	21.6%	34.2%	12.5%

(17) My child uses a few sounds but does not make many different sounds	24.5%	20.1%	37%	18.5%

**Table 7:** Percentages of parents on question of imitation skills.

	Always	Frequently	Sometimes	Never
(35) It is hard for my child to imitate a word that i say	27.6%	18.2%	36.4%	17.9%

**Table 8:** Percentages of parents on question of prosody and rhythm.

	Always	Frequently	Sometimes	Never

(21) My child speaks rapidly	5.3%	10.3%	27.6%	56.7%

(30) My child prolongs vowel sounds	12.5%	5%	35.4%	47%

**Table 9:** Percentages of parents on question of struggle when speaking.

	Always	Frequently	Sometimes	Never

(20) My child seems to be struggling so hard to say words and sounds	18.2%	16.3%	45.1%	20.4%

**Table 10:** Percentages of parents on question of hearing loss.

	Always	Frequently	Sometimes	Never

(23) My child has difficulty hearing	3.8%	2.5%	3.4%	90.3%

## Appendix

### Down Syndrome Speech Intelligibility Survey

We are trying to learn more about the factors that influence whether people can understand your child's speech. The purpose of this survey is to support our efforts at understanding the effectiveness of clinical services. The results will advance our ability to treat speech-related problems. We hope to share these findings with families and professionals through conference presentations and journal articles. Of course, no names or identifying data will be included in these reports. The few minutes you take to complete this form will be a tremendous help in our efforts to better understand and treat speech-related problems for children with Down syndrome. Thank you for taking the time to complete the survey.

Today's Date —
Child's Name —
Child's Gender
  □ Female
  □ Male
Child'd Birthdate (Month/Day/Year)
My child began to speak at about
  □ 2 years
  □ 3 years
  □ 4 years
  □ 5 years
  □ After 5 years
On a scale of 1 to 10, where 1 is completely unintelligible and 10 is completely intelligible, how would you rate your child's speech? —
Have you been told that your child has oral motor difficulties?
  □ Yes
  □ No
Have you been told that your child has apraxia or dyspraxia?
  □ Yes
  □ No
My child communicates by using
  □ Speech
  □ Pictures/Photos
  □ Sign Language
  □ High Tech Communication System
  □ Communication Board
  □ Other ( )

*(For each question, please check only ONE answer)*

1. People who know my child well have difficulty understanding his/her speech
  □ always
  □ frequently
  □ sometimes
  □ never
2. People who first meet my child have difficulty understanding his/her speech
  □ always
  □ frequently
  □ sometimes
  □ never
3. My child communicates primarily by using speech
  □ always
  □ frequently
  □ sometimes
  □ never
4. When someone can't understand my child's speech, family members interpret for him or her
  □ always
  □ frequently
  □ sometimes
  □ never
5. In infancy, my child made cooing sounds (single sounds)
  □ always
  □ frequently
  □ sometimes
  □ never
6. In infancy, my child babbled strings of sounds
  □ always
  □ frequently
  □ sometimes
  □ never
7. My child had difficulty sucking and swallowing liquids in infancy
  □ always
  □ frequently
  □ sometimes
  □ never
8. My child had feeding difficulties when s/he started eating solid foods
  □ always
  □ frequently
  □ sometimes
  □ never
9. My child currently has difficulties with swallowing liquids
  □ always
  □ frequently
  □ sometimes
  □ never
10. My child currently has difficulties with feeding/eating
  □ always
  □ frequently
  □ sometimes
  □ never
11. My child had low tone in the muscles of the face (lips, tongue, cheeks) in infancy
  □ always
  □ frequently
  □ sometimes
  □ never
12. My child currently has low tone in the muscles of the face (lips, tongue, cheeks)
  □ always
  □ frequently
  □ sometimes
  □ never
13. My child was late (delayed) in beginning to speak
  □ always
  □ frequently
  □ sometimes
  □ never
14. My child makes the same speech errors consistently
  □ always
  □ frequently
  □ sometimes
  □ never
15. Sometimes, my child can say a word but at other times, my child has difficulty saying the same word
  □ always
  □ frequently
  □ sometimes
  □ never
16. My child is understandable when s/he says single words, but has greater difficulty in conversation
  □ always
  □ frequently
  □ sometimes
  □ never
17. My child uses a few sounds, but does not make many different sounds
  □ always
  □ frequently
  □ sometimes
  □ never
18. My child can sing the words in songs more clearly than s/he can say them when speaking
  □ always
  □ frequently
  □ sometimes
  □ never
19. My child shows very slow improvement in speech therapy
  □ always
  □ frequently
  □ sometimes
  □ never
20. My child seems to be struggling so hard to say words and sounds
  □ always
  □ frequently
  □ sometimes
  □ never
21. My child speaks rapidly
  □ always
  □ frequently
  □ sometimes
  □ never
22. My child has fluency (stuttering-like) difficulties when speaking
  □ always
  □ frequently
  □ sometimes
  □ never
23. My child has difficulty hearing
  □ always
  □ frequently
  □ sometimes
  □ never
24. My child has more difficulty saying longer words than shorter words
  □ always
  □ frequently
  □ sometimes
  □ never
25. My child has more difficulty speaking when s/he is using longer phrases or sentences
  □ always
  □ frequently
  □ sometimes
  □ never
26. My child has difficulty saying some consonant sounds
  □ always
  □ frequently
  □ sometimes
  □ never
27. My child has difficulty saying some vowel sounds
  □ always
  □ frequently
  □ sometimes
  □ never
28. My child often reverses sounds in words (e.g., aminal for animal)
  □ always
  □ frequently
  □ sometimes
  □ never
29. My child has difficulty with the rhythm of speech (speech sounds choppy, or sometimes slow and sometimes fast)
  □ always
  □ frequently
  □ sometimes
  □ never
30. My child prolongs vowel sounds
  □ always
  □ frequently
  □ sometimes
  □ never
31. My child leaves out sounds in words
  □ always
  □ frequently
  □ sometimes
  □ never
32. My child leaves out syllables in words
  □ always
  □ frequently
  □ sometimes
  □ never
33. My child's speech sounds hypernasal (as if it's coming through his/her nose)
  □ always
  □ frequently
  □ sometimes
  □ never
34. My child talks less with people outside of the circle of friends and family
  □ always
  □ frequently
  □ sometimes
  □ never
35. It is hard for my child to imitate a word that I say
  □ always
  □ frequently
  □ sometimes
  □ never
36. My child's speech is easier to understand when s/he is saying familiar words
  □ always
  □ frequently
  □ sometimes
  □ never
37. My child understands more than s/he can say
  □ always
  □ frequently
  □ sometimes
  □ never
38. My child may unexpectedly say a word or phrase perfectly, but then s/he can't repeat it
  □ always
  □ frequently
  □ sometimes
  □ never
39. My child has difficulty with grammar
  □ always
  □ frequently
  □ sometimes
  □ never
40. My child is frustrated when people don't understand what s/he is saying.
  □ always
  □ frequently
  □ sometimes
  □ never
